# Effect of Different Laser Wavelengths on Periodontopathogens in Peri-Implantitis: A Review of In Vivo Studies

**DOI:** 10.3390/microorganisms7070189

**Published:** 2019-06-29

**Authors:** Katarzyna Świder, Marzena Dominiak, Kinga Grzech-Leśniak, Jacek Matys

**Affiliations:** 1 Dental Prosthodontics Clinic, Medical University of Wroclaw, 50-425 Wroclaw, Poland; 2 Dental Surgery Department, Medical University of Wroclaw, 50-425 Wroclaw, Poland; 3 Private Dental Practice Ka-dent, Lipowa 18, 67-400 Wschowa, Poland

**Keywords:** laser, peri-implantitis, periodontopathogens, antimicrobial photodynamic therapy, decontamination, systematic review

## Abstract

Nowadays, many studies are examining the effectiveness of dental lasers in the treatment of peri-implantitis; however, most of them only report periodontal parameter changes. The authors of this review tried to address the question: “What is the effect of different laser wavelengths on oral bacteria that cause peri-implantitis?” An electronic search of PubMed and Cochrane Central Register of Controlled Trials was performed. The following search terms were used: (peri-implantitis OR periimplantitis) OR/AND (microbial OR microbiologic) AND (laser OR Er:YAG OR erbium OR diode OR Nd:YAG OR neodymium-doped OR Er,Cr:YSGG OR chromium-doped). Initially, 212 studies were identified. After screening the titles and abstracts and excluding studies according to predefined inclusion criteria, seven publications were included in the review. Three studies about the effect of aPDT (antimicrobial photodynamic therapy) reported a decrease in the different bacterial strains associated with peri-implantitis, e.g., *A. actinomycetemcomitans*, *P. gingivalis*, *P. intermedia*, *T. denticola*, *T. forsythia*, *F. nucleatum*, and *C. rectus*. Two studies showed that the high-power diode laser may have some effect on peri-implant pathogens. Two articles about the Er:YAG laser reported a lowering in the count of oral pathogens; however, it was hard to determine if this was due to the use of the laser. aPDT has the ability to decrease the count of peri-implant pathogens, whereas Er:YAG laser application shows no significant effect on oral bacteria in the long term.

## 1. Introduction

Endosseous implants are a widely chosen prosthetic rehabilitation treatment for missing teeth [1,2]. However, studies report that up to 56% of implant patients and even 43% of implant sites can be ailed by the peri-implant inflammatory process, known as peri-implantitis [3]. Peri-implantitis is described as inflammation of an implant supporting tissues in association with bone loss, which if left untreated, can result in the destruction of the bone [3].

**Figure 1:**
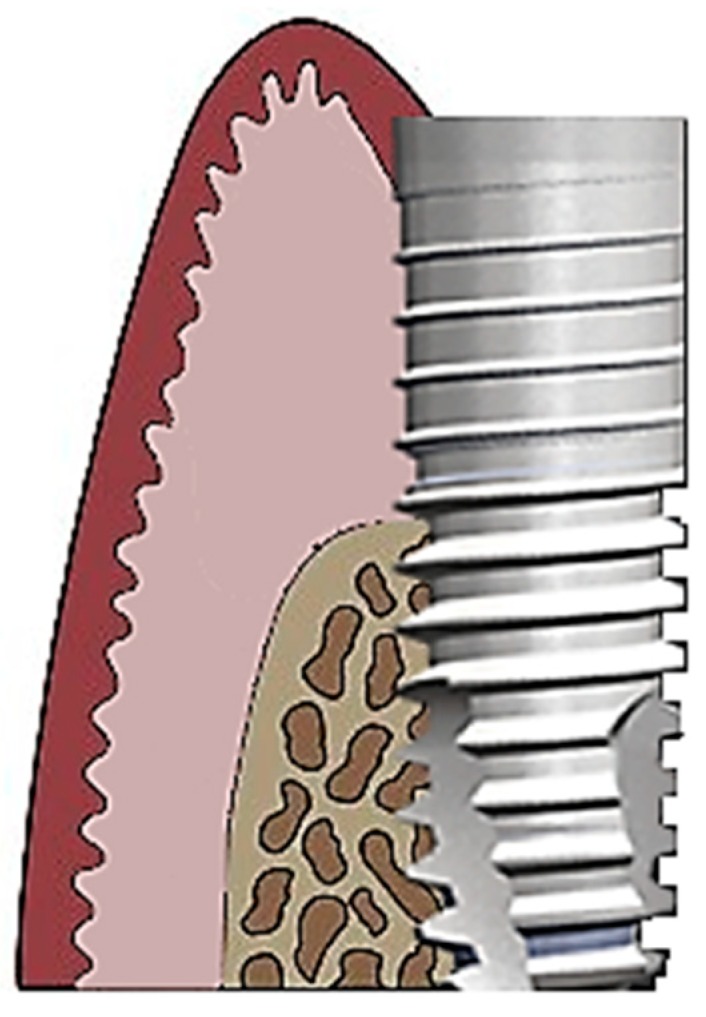
Schematic diagram of peri-implant tissue.

The etiology of peri-implant disease is diverse. Peri-implantitis is reported to occur more frequently in nicotine smokers [4] and in patients with periodontitis [5,6]. Nonetheless, the presence of microorganisms is fundamental for the development of the infection [7,8]. As studies have shown, [7,8] peri-implantitis harbors high levels of diverse periodontal pathogens, predominantly gram-negative species. This is also because peri-implant tissue is more complex then periodontal tissue. Peri-implant tissue is characterized by a lack of the periodontal ligament, cementum, and has altered connective tissue fibers (Figure 1).

The current principles of peri-implantitis treatment are based on results established from periodontal disease therapies, and they can be divided into mechanical and/or chemical treatments [9]. Mombelli et al. [8] suggested that decontaminating the implant surface, reducing or removing periodontal pockets, re-osseointegration, and proper oral hygiene should be employed in peri-implantitis treatment. In mechanical modalities (subgingival debridement, conventional and ultrasonic scaling, carbon fiber curettes), [8,9] the main goal is the removal of biofilm from implant surfaces. However, these methods can damage the surface of implants, which can be seen in scanning electron microscopy, without effectively decontaminating the surface [10,11] or producing a significant pocket depth improvement [11]. In addition, various systemic and local chemical antimicrobial agents (citric acid, chlorhexidine, hydrogen peroxide application) [9,12] and antibiotics have been introduced for the treatment of peri-implantitis [8,13,14]. These agents complement mechanical treatment and result in more effective eradication of periodontopathogens. Nonetheless, antibiotic modalities are not free from disadvantages and can cause increases in antibiotic resistant periodontopathogens, negative systemic reactions, and immunosuppression in patients [8], whereas chemical agents can cause tissue damage or irritation [10,13].

An alternative antimicrobial treatment without the complications listed above is laser-assisted therapy. Laser irradiation targets pathogens locally; thus, no systemic reactions exist. Various in vitro studies report that some laser wavelengths can not only eliminate bacteria but also bacterial toxins and lipopolysaccharides (LPS) [15,16]. In periodontal diseases, lasers can be used as a separate treatment or in addition to conventional scaling and root planning [17,18,19].

Nowadays, a variety of lasers are used in dental procedures as innovative therapeutic techniques that reduce bleeding, swelling, and pain [20,21,22,23,24,25]. The most used lasers are the diode and the Er:YAG laser [20]. They can be employed in both non-surgical and surgical procedures [21]. The diode laser is characterized by a hemostatic effect, the promotion of healing, and its use in sulcular debridement [21]. The Er:YAG laser has the capability to be absorbed by water molecules and therefore results in minimal thermal damage and tissue carbonization [21]. Thus, bacteria are eliminated without overheating the surrounding tissues. Additionally, some studies report that the Er:YAG laser is capable of removing plaque and calculus even from rough implant surfaces [21,26,27].

The aim of the present study was to evaluate the bactericidal potential of laser application in patients with peri-implantitis.

## 2. Materials and Methods

### 2.1. Focused Question

A growing body of researchers is examining the clinical effectiveness of dental lasers in the treatment of peri-implantitis. However, most of the studies mainly engage with changes in periodontal parameters, less often in oral periodontal pathogens, and most them are in vitro studies. Therefore, the addressed focused question in this paper was “What is the effect of different laser wavelengths on oral bacteria that cause peri-implantitis?”

### 2.2. Protocol

The text of the review was structured in accordance with guidelines from PRISMA [28] and the Cochrane Handbook of Systematic Reviews of Interventions [29]. A detailed description of the study protocol is included in this paper (Table 1).

### 2.3. Eligibility Criteria

Studies were considered acceptable for inclusion in the review if they met the following criteria:

- Studies involving human subjects;
- Patients with peri-implantitis;
- Surgical or non-surgical use of dental lasers in the treatment of peri-implantitis;
- Evaluated changes in specified oral bacterial profiles before and after the laser treatment;
- Prospective case series;
- Non-randomized controlled clinical trials (NRS); and
- Randomized controlled clinical trials (RCT).

The exclusion criteria the reviewers agreed upon were as follows (Table 1):

- Animal studies;
- In vitro studies;
- Review articles;
- No full-text accessible; or
- Duplicated publications.

No restrictions were applied with regard to the year of publication or to the language of the study.

### 2.4. Information Sources, Search Strategy, and Study Selection

An electronic search of PubMed and the Cochrane Central Register of Controlled Trials (CENTRAL) databases was conducted on 5 April, 2019. To review the data available on the subject of interest, the following search terms were used: (peri-implantitis OR periimplantitis) OR/AND (microbial OR microbiologic) AND (laser OR Er:YAG OR erbium OR diode OR Nd:YAG OR neodymium-doped OR Er,Cr:YSGG OR chromium-doped) (Table 1). The search was limited to human subjects and studies that adhered to other eligibility criteria. The references of all selected full-text articles and related reviews were scanned. Only papers with available or accessible full-text versions were considered. If required, an attempt was made to contact the corresponding authors of unpublished or missing data. Screening was performed by each author and the acquired data were compared.

### 2.5. Data Collection Process, Data Items

Two reviewers independently extracted data from papers that met the inclusion criteria. The following data were used: first author, title, year of publication, study design, laser type, laser parameters, and changes in specified oral bacterial profiles before and after treatment. Extracted data were entered into a standardized Excel file.

### 2.6. Risk of Bias in Individual Studies

In the initial study selection, to minimize the potential for reviewer bias, each author screened titles and abstracts independently. The level of agreement between reviewers was determined by the Cohen *k* test. Any disagreement about the inclusion or exclusion of a study was resolved by discussion.

### 2.7. Quality Assessment

The methodological quality of each included study was evaluated independently by two blinded reviewers. The study design, implementation, and analysis were based on the following criteria: population representativeness in the treatment group (average of the population), demonstration that the outcomes were not present at the start of the treatment, comparability of the baseline and the outcome parameters, accuracy of the microbial genome evaluation technique, randomization, adequate follow-up (for outcomes to occur), and acceptable follow-up loss (complete follow-up, subjects lost to follow-up unlikely to introduce bias) (Table 2). The descriptive information about the studies was graded. The score range was from 0 to 6 points, with a higher score indicating a higher study quality. Any disagreements were resolved through discussion until reaching a consensus.

### 2.8. Risk of Bias Across Studies

After the scores of each study were calculated, an overall estimate of the risk of bias (low, moderate, or high) was made for each publication, as recommended in the Cochrane Handbook for Systematic Reviews of Interventions [29].

## 3. Results

### 3.1. Study Selection

Initially, 212 studies were identified. After screening of the titles and abstracts, 177 studies were excluded. Forty-eight studies were selected for thorough full-text screening, from which 41 were excluded according to predefined inclusion criteria [35,36,37,38,39,40,41,42,43,44,45,46,47,48,49,50,51,52,53,54,55,56,57,58,59,60,61,62,63,64,65,66,67,68,69,70,71,72,73,74,75] (Table 3). Finally, seven publications were included in the review [14,15,30,31,32,33,76] (Table 4).

### 3.2. General Characteristics of the Included Studies

Seven articles reporting four randomized controlled trials [14,30,32,33] and three prospective case series [31,34,76] were included in this review (Table 4). Two studies evaluated the photothermal effect of the diode laser [30,77], two evaluated the Er:YAG laser [32,34] and three evaluated aPDT (antimicrobial photodynamic therapy) [14,31,76] on peri-implant pathogens. Various bacterial profiles were assessed in the articles. Also, the follow-up period differentiated from right after the therapy to up to 2 years (Table 5).

### 3.3. Results of Individual Studies

In the study by Dörtbudak et al. [76], the authors reported that the aPDT reduced the bacterial counts of *A. actinomycetemcomitans* (A.a.), *P. gingivalis* (P.g.), and *P. intermedia* (P.i.). Also, Bassetti et al. [14] found that mechanical debridement and additional aPDT (TBO dye) seem to be able to decrease the total bacterial count, but the difference was reported to be of no significance. Caccianiga et al. [31] reported a medium decrease of most periodontal pathogens, including A.a., P.g., *T. denticola* (T.d.), *T. forsythia* (T.f.), *F. nucleatum* (F.n.), and *C. rectus* (C.r.) but excluding *E. corrodens* (E.c.), that increased in count after aPDT (3% hydrogen peroxide). Moreover, in the research by Birang et al. [30], diode laser irradiation of the implant site decreased the counts of P.g. and A.a. The authors also reported significantly decreased A.a., T.f., and P.g. in the test group that used a photosensitizer. These findings correspond to the study by Arisan et al. [33] who concluded that diode laser application reduced the total bacterial count, but the difference was stated to be of no significance.

Yoshino et al. [34], in their study on the Er:YAG laser, reported eradication of oral pathogens. Additionally, Persson et al. [32] showed lower bacterial counts in the laser-treated group for *F. nucleatum naviforme* and *F. nucleatum nucleatum* at 1 month after treatment and in comparison to baseline levels. However, at 3 months and at the final examination at 6 months, counts of bacteria increased (Table 6).

### 3.4. Synthesis of Results

The studies included in the review varied in terms of the following features: laser parameters, evaluated periodontal pathogens (Table 4), follow-up period, and microbial genome evaluation (Table 5). Collected features were analyzed and put into an Excel file (Table 6).

The authors of the review paid special attention only to significant microbiological changes in the bacterial counts of each study at each follow-up period. It was impossible to analyze each bacterial count since some studies did not include that information. Therefore, the authors decided to focus on the changes (lower, higher) in relation to the baseline, accentuating significant variables (Table 6).

### 3.5. Risk of Bias Across Studies

The articles by Persson et al. [32] and Birang et al. [30] reported blinding of the patients and examiner and random allocation of the patients by a computer program; therefore, the studies were considered to have a low risk of bias. Another two were RCTs and had some above information missing and were reported to be at a medium risk of bias [14,33]. Since three studies were prospective case series [31,34,76], they were considered to be at a high risk of bias (Table 7).

## 4. Discussion

The findings of this review show that laser application in peri-implantitis may decontaminate the implant surface and eradicate periodontal pathogens in some cases. The seven papers included to this review can be divided according to the laser treatment modalities used. Two studies evaluated the photothermal effect of the diode laser [30,33], two evaluated the Er:YAG laser [32,34] and three evaluated antimicrobial photodynamic therapy [14,31,76] on the eradication of peri-implant pathogens.

In the studies of Arisan et al. [33] and Birang et al. [30], the authors used high-power diode lasers to eradicate the bacterial biofilm from the implant titanium surface due to the photothermal effect. The use of high-power diode lasers on tissues is inextricably linked with the increase of their temperature, which, in turn, causes several changes in the structure of tissues through protein denaturation, microbial elimination, water evaporation, coagulation, and even melting [17,18]. The first changes in the structure of the oral soft tissue begin when its temperature was increased to 42 °C (ΔT = 5 °C), and then changes occurred within the cell membranes [78]. In the study by Arisan et al. [33], mechanical therapy and additional diode laser application decreased the total bacterial count on the affected peri-implant zone. The author measured insignificant differences in the results of decreasing of A.a., P.g., T.d., T.f., C.r., E.c. species after a 1 month follow-up period in contrast to the control group. In turn, the research by Birang et al. [30] showed that diode laser (810 nm, 300 mW, 30 s) irradiation of the implant site decreased the count of P.g., and differences in A.a. reached the significance threshold. Moreover, the authors reported significant decreases in the A.a., T.f., and P.g. strains after 3 months in the test group that additionally used infracyanine green. However, there were no significant differences between the laser modalities (with or without infracyanine green).

Antimicrobial photodynamic therapy was applied for peri-implantitis treatment in the studies of Dörtbudak et al. [76], Bassetti et al. [14], and Caccianiga et al. [31]. During this process, a combination of nontoxic photosensitizing dyes with appropriate wavelengths of light led to the production of reactive oxygen species. The reactive oxygen species produced with aPDT had high killing potential against bacteria, fungi, and viruses [33]. In the study by Dörtbudak et al. [76], the authors evaluated the culture counts for *A. actinomycetemcomitans*, *P. gingivalis*, and *P. intermedia*. It was found that aPDT reduced the bacterial counts measured immediately after therapy by 2 logs. These findings correspond to Caccianiga et al. [31], who reported a medium decrease in most periodontal pathogens (A.a., P.g., T.d., T.f., F.n., C.r.) excluding E.c. which increased in count after a 6 month follow-up period. Also, in the study by Bassetti et al. [14], mechanical debridement and additional aPDT application reduced the total bacterial counts on affected peri-implant sulcus. The counts of P.g. and T.f. decreased significantly from baseline to 6 months following aPDT and after 12 months in the local antibiotics group. The authors concluded that aPDT seems to be an alternative approach to local antibiotics for non-surgical therapy of peri-implantitis.

Two articles assessed the influence of the Er:YAG laser on oral cavity microbes associated with peri-implantitis [32,34]. The Er:YAG laser has the highest absorption coefficient in water, which is the main component of vital organisms. This physical property influences the ability of the wavelength to damage of the water-rich cells and constitutes the significant killing potential of this laser. [79] Yoshino et al. [34], in their study, reported on the eradication of oral pathogens; however, the laser application was preceded by a systemic antibiotic protocol (amoxycilin, metronidazol); therefore, it is impossible to conclude the real reason for the bacteria count decrease. In turn, Persson et al. [32] showed lower bacterial counts in the laser-treated group for *F. nucleatum naviforme* and *F. nucleatum nucleatum* at 1 month after treatment and in comparison to baseline levels. However, at 3 months and at the final examination at 6 months, the counts of bacteria increased. It should be highlighted that the study conducted by Person et al. [32] was a non-surgical approach to the treatment of peri-implantitis. The threads and rough surfaces of implants are complicated to manage by non-flap methods; thus, this could explain the lack of reduction in the total bacterial amount in the study.

More studies with bigger populations of patients are needed to determine the use of dental lasers in bacteria decontamination.

## 5. Conclusions

It can be concluded that a high-power diode laser may have some effect on peri-implant pathogens causing peri-implantitis, whereas Er:YAG laser application shows no significant effect on oral bacteria in the long term.

aPDT has the ability to reduce the total count of the different bacterial strains associated with peri-implantitis, e.g., *A. actinomycetemcomitans*, *P. gingivalis*, *P. intermedia*, *T. denticola*, *T. forsythia*, *F. nucleatum*, and *C. rectus*.

## Figures and Tables

**Table 1:** Systematic Search Strategy.

Focused Question	What is the Effect of Different Laser Wavelengths on Oral Bacteria that Cause Peri-Implantitis?
Search strategy	
Population	Patients diagnosed with peri-implantitis
Intervention or exposure	Surgical or non-surgical laser treatment
Comparison	Changes in oral bacterial profiles before and after treatment
Outcome	Changed oral bacterial profiles or the number of specified bacteria
Search combination	(peri-implantitis OR periimplantitis) OR/AND (microbial OR microbiologic) AND (laser OR Er:YAG OR erbium OR diode OR Nd:YAG OR neodymium-doped OR Er,Cr:YSGG OR chromium-doped)
Electronic database search	PubMed, Cochrane Central Register of Controlled Trials (CENTRAL)
Selection criteria	
Inclusion criteria	Studies involving human subjects Patients with peri-implantitis Surgical or non-surgical use of dental lasers in the treatment of peri-implantitis Evaluated changes in specified oral bacterial profiles before and after the laser treatment Prospective human case series Non-randomized controlled clinical trials (NRS) Randomized controlled clinical trials (RCT)
Exclusion criteria	Animal studies In vitro studies Review articles No full-text accessible Duplicated publications

**Table 2:** Quality assessment of studies.

Criteria	First Author					
	Birang et al. [30]	Caccianiga et al. [31]	Persson et al. [32]	Arisan et al. [33]	Yoshino et al. [34]	Bassetti et al. [14]
Population representativeness in the treatment group (average of the population)	1	1	1	1	0	1
Comparability of the baseline and the outcome parameters	1	1	1	1	1	1
Accuracy of the microbial genome evaluation technique	1	1	0	0	0	1
Randomization	1	0	1	0	0	0
Adequate follow-up (for outcomes to occur)	1	1	1	1	1	1
Acceptable follow-up loss (complete follow-up, subjects lost to follow-up unlikely to introduce bias)	1	1	1	1	1	1
Total	6	5	5	4	3	5

**Table 3:** Reasons for exclusion of studies.

First Author	Year of Publication	Reason for Exclusion
Salaria et al. [35]	2018	No bacterial profile evaluated
Karimi et al. [36]	2016	No bacterial profile evaluated
Schwarz et al. [37]	2011	No bacterial profile evaluated
Schwarz et al. [38]	2017	No bacterial profile evaluated
Schwarz et al. [39]	2015	No bacterial profile evaluated
Schwarz et al. [40]	2004	Systematic review
Scarano et al. [41]	2016	An in vitro study
Pommer et al. [42]	2016	No bacterial profile evaluated
Norton [43]	2017	No bacterial profile evaluated
Lerario et al. [44]	2016	No bacterial profile evaluated
Hegazy et al. [45]	2016	No bacterial profile evaluated
John et al. [46]	2017	No bacterial profile evaluated
Valente et al. [47]	2018	No bacterial profile evaluated
Romeo et al. [48]	2016	No bacterial profile evaluated
Al Amri et al. [49]	2016	No bacterial profile evaluated
Abduljabbar et al. [50]	2017	No bacterial profile evaluated
Larsen et al. [51]	2017	An in vitro study
Nicholson et al. [52]	2017	No bacterial profile evaluated
Spadari et al. [53]	2010	No full-text accessible
Bombeccari et al. [54]	2013	No bacterial profile evaluated
Chambrone et al. [55]	2018	Systematic review
Esposito et al. [56]	2008	Systematic review
Ashnagar et al. [57]	2014	Systematic review
Papadopoulos et al. [58]	2015	No bacterial profile evaluated
Renvert et al. [59]	2008	Systematic review
Renvert et al. [60]	2011	No bacterial profile evaluated
Yan et al. [61]	2015	Systematic review
Natto et al. [62]	2015	Systematic review
Smeets et al. [63]	2014	Systematic review
Kotsakis et al. [64]	2014	Systematic review
Figuero et al. [65]	2000	Systematic review
Suárez-López Del Amo et al. [66]	2016	Systematic review
Alshehri et al. [67]	2016	Systematic review
Ghanem et al. [68]	2016	Systematic review
Mizutani et al. [69]	2000	Systematic review
Mahato et al. [70]	2016	Systematic review
Al Habashneh et al. [71]	2015	Systematic review
Subramani et al. [72]	2012	Systematic review
Rajesh et al. [73]	2011	Systematic review
Gonçalves at al. [74]	2010	Systematic review
Kotsovilis at al. [75]	2008	Systematic review

**Table 4:** General characteristics of the included studies.

First Author	Study Design (No. of Subjects)	Laser Type	Laser Parameters	Evaluated Bacteria
Birang et al. [30]	RCT	diode	810 nm, 300 mW, 30 s per site, large-area handpiece (transgingival) or bulb fiber (intra-pocket) or bare fiber (granulation tissues), irradiation repeated after 2 weeks	*Aggregatibacter actinomycetemcomitans*, *Porphyromonas gingivalis, Prevotella intermedia*, *Treponema denticola*, *Tannerella forsythia*
Caccianiga et al. [31]	prospective case series	diode (aPDT)	2.5 W, 0.5 W (mean power), 10 kHz, T-on 20 us, T-off 80 us, 60 s per site, 400 micron fiber, periodontal and peri-implant pocket site, irradiation repeated after 15 days and then for the next 3 months every 20 days, 3% hydrogen peroxide	*Aggregatibacter actinomycetemcomitans*, *Porphyromonas gingivalis*, *Treponema denticola*, *Tannerella forsythia*, *Fusobacterium nucleatum*, *Campylobacter rectus*, *Eikenella corrodens*
Persson et al. [32]	RCT	Er:YAG	100 mJ/pulse, 10 Hz (12.7 J/cm), cone-shaped sapphire tip, parallel mode, pocket site	74 specimens (*Campylobacter showae*, *Capnocytophaga ochracea*, *P. melaninogenica*, *S. anaerobius*, *S. hae- molyticus*, *S.intermedius*, and *S. mutans*)
Arisan et al. [33]	RCT	diode	810 nm (energy density, 3 J/cm^2^; power density, 400 mW/cm^2^; energy 1.5 J; spot diameter, 1 mm), 60 s per site, pulsed mode, power level of 1 W, 400 um optical fiber tip, peri-implant pocket area	20 specimen (*Tannerella forsythia*, *Treponema denticola*, *Porphyromonas gingivalis*, *Campylobacter rectus*, *Prevotella intermedia*, *Peptostreptococcus micros*, *Fusobacterium nucleatum*, *Eubacterium nodatum*, *Streptococcus constellatus* group, *Campylobacter gracilis*, *Prevotella nigrescens*)
Yoshino et al. [34]	prospective case series	Er:YAG	150 mJ (10 ps), 40 mJ (10 pps), 70 mJ (25 pps), straight tip (bone area), side tip (implant area), straight-and-side (gingival sulcus area)	*Aggregatibacter actinomycetemcomitans*, *Porphyromonas gingivalis*, *Prevotella intermedia*, *Treponema denticola*, *Tannerella forsythia*
Bassetti et al. [14]	RCT	diode (aPDT)	660 nm, 100 mW, 10 s per site, peri-implant pocket area, irradiation repeated after 1 week toluidine blue O dye (TBO)	*Aggregatibacter actinomycetemcomitans*, *Porphyromonas gingivalis*, *Prevotella intermedia*, *Treponema denticola*, *Tannerella forsythia*, *Fusobacterium nucleatum*, *Campylobacter rectus*, *Capnocytophaga gingivalis*, *Parvimonas micra*, *Eubacterium nodatum*, *Eikenella corrodens*
Dörtbudak et al. [76]	prospective case series	diode (aPDT)	690 nm, 60 s per site, implant and per-implant pocket site TBO	*Aggregatibacter actinomycetemcomitans*, *Porphyromonas gingivalis*, *Prevotella intermedia*

**Table 5:** Follow-up and microbial test assessment of the included studies.

First author	Subject Groups	Microbial Genome Evaluation	Follow-Up
Birang et al. [30]	Control (mechanical debridement + diode laser), test (mechanical debridement + diode laser EmunDo)	real-time polymerase chain reaction (RT-PCR) technique	3 months
Caccianiga et al. [31]	Only one group (aPDT Oxylaser)	real-time polymerase chain reaction (RT-PCR) technique	6 months
Persson et al. [32]	Test 1 (Er:YAG laser), test 2 (air-abrasive device)	DNA–DNA hybridization method	6 months
Arisan et al. [33]	Control (mechanical debridement), test (mechanical debridement + diode laser)	polymerase chain reaction (PCR) technique	6 months
Yoshino et al. [34]	Only one group (Er:YAG laser)	polymerase chain reaction (PCR) technique	2 years
Bassetti et al. [14]	Control (mechanical debridement), test (mechanical debridement + aPDT)	real-time polymerase chain reaction (RT-PCR) technique	12 months
Dörtbudak et al. [76]	Control (no treatment), test 1 (dye), test 2 (dye + laser—aPDT)	gram staining, colony morphology, positive catalase reaction, BANA, hydrolytic activity, a-glucosidase activity, ß-galactosidase, esculin hydrolysis and indole test	Right after the therapy

**Table 6:** Results of individual studies.

Periodontal Pathogens	Follow-Up	First Author						
		Birang et al. [30]	Caccianiga et al. [31]	Persson et al. [32]	Arisan et al. [33]	Yoshino et al. [34]	Bassetti et al. [14]	Dörtbudak et al. [76]
*Aggregatibacter actinomycetemcomitans*	Right after the therapy	ne	ne	ne	ne	ne	ne	L *
	1 month	ne	ne	H	L	ne	ne	ne
	3 months	L *	ne	H	ne	ne	L *	ne
	6 months	ne	L *	H	ne	ne	L *	ne
	1 year	ne	ne	ne	ne	ne	L *	ne
	2 years	ne	ne	ne	ne	L	ne	ne
*Porphyromonas gingivalis*	Right after the therapy	ne	ne	ne	ne	ne	ne	L *
	1 month	ne	ne	H	L	ne	ne	ne
	3 months	L *	ne	H	ne	ne	L *	ne
	6 months	ne	L *	H	ne	ne	L *	ne
	1 year	ne	ne	ne	ne	ne	L *	ne
	2 years	ne	ne	ne	ne	L	ne	ne
*Prevotella intermedia*	Right after the therapy	ne	ne	ne	ne	ne	ne	L *
	1 month	ne	ne	H	0	ne	ne	ne
	3 months	L	ne	H	ne	ne	L *	ne
	6 months	ne	L *	H	ne	ne	L *	ne
	1 year	ne	ne	ne	ne	ne	L *	ne
	2 years	ne	ne	ne	ne	L	ne	ne
*Treponema denticola*	Right after the therapy	ne	ne	ne	ne	ne	ne	ne
	1 month	ne	ne	H	L	ne	ne	ne
	3 months	L	ne	H	ne	ne	L *	ne
	6 months	ne	L *	H	ne	ne	L *	ne
	1 year	ne	ne	ne	ne	ne	L *	ne
	2 years	ne	ne	ne	ne	L	ne	ne
*Tannerella forsythia*	Right after the therapy	ne	ne	ne	ne	ne	ne	ne
	1 month	ne	ne	H	L	ne	ne	ne
	3 months	L *	ne	H	ne	ne	L *	ne
	6 months	ne	L *	H	ne	ne	L *	ne
	1 year	ne	ne	ne	ne	ne	L *	ne
	2 years	ne	ne	ne	ne	L	ne	ne
*Fusobacterium nucleatum*	Right after the therapy	ne	ne	ne	ne	ne	ne	ne
	1 month	ne	ne	L *	0	ne	ne	ne
	3 months	ne	ne	H	ne	ne	L *	ne
	6 months	ne	L *	H	ne	ne	L *	ne
	1 year	ne	ne	ne	ne	ne	L *	ne
	2 years	ne	ne	ne	ne	ne	ne	ne
*Campylobacter rectus*	Right after the therapy	ne	ne	ne	ne	ne	ne	ne
	1 month	ne	ne	H	L	ne	ne	ne
	3 months	ne	ne	H	ne	ne	L *	ne
	6 months	ne	L *	H	ne	ne	L	ne
	1 year	ne	ne	ne	ne	ne	L	ne
	2 years	ne	ne	ne	ne	ne	ne	ne
*Eikenella corrodens*	Right after the therapy	ne	ne	ne	ne	ne	ne	ne
	1 month	ne	ne	H	L	ne	ne	ne
	3 months	ne	ne	H	ne	ne	L *	ne
	6 months	ne	H	H	ne	ne	L	ne
	1 year	ne	ne	ne	ne	ne	L	ne
	2 years	ne	ne	ne	ne	ne	ne	ne

L = lower than baseline; H = higher than baseline; ne = not evaluated; * = significant difference; 0 = no changes.

**Table 7:** Risk of bias across studies.

First Author	Randomization	Blinding Examiner	Blinding Patients	Statistical Methods
Birang et al. [30]	Program software	yes	yes	SPSS 20, Kruskal–Wallis test, Friedman’s and Wilcoxon tests, Wilcoxon test
Caccianiga et al. [31]				
Persson et al. [32]	Program software	yes	yes	Kruskal–Wallis test, Mann–Whitney U tests, Wilcoxon test, Spearman rank correlation, x2 analysis
Arisan et al. [33]	Not described	Not described	Not described	D’Agastino Pearson Omnibus Normality test, Sidak’s test, Fisher’s exact test, McNemar Test
Yoshino et al. [34]				
Bassetti et al. [14]	Not described	yes	Not described	SD, Student‘s t-test, Wilcoxon test, Chi-square test, Mann–Whitney U-test, Fisher’s exact test
Dörtbudak et al. [76]				Tukey Student test
